# Congenital and Postnatal CMV and EBV Acquisition in HIV-Infected Zimbabwean Infants

**DOI:** 10.1371/journal.pone.0114870

**Published:** 2014-12-18

**Authors:** Hlanai Gumbo, Bernard Chasekwa, James A. Church, Robert Ntozini, Kuda Mutasa, Jean H. Humphrey, Andrew J. Prendergast

**Affiliations:** 1 Zvitambo Institute for Maternal and Child Health Research, Harare, Zimbabwe; 2 Centre for Paediatrics, Blizard Institute, Queen Mary University of London, London, United Kingdom; 3 Department of International Health, Johns Hopkins Bloomberg School of Public Health, Baltimore, MD, United States of America; University of British Columbia, Canada

## Abstract

**Background:**

HIV-infected infants in sub-Saharan Africa have rapid disease progression. We hypothesized that co-infection with cytomegalovirus (CMV) or Epstein Barr virus (EBV) increases mortality in HIV-infected infants.

**Methods:**

257 antiretroviral therapy-naïve HIV-infected Zimbabwean infants were tested for CMV and EBV at 6 weeks of age by real-time PCR; if positive, birth samples were retrieved where available to distinguish congenital and postnatal infection. The impact of co-infection on mortality through 6 months was estimated using Kaplan-Meier and Cox proportional hazards methods.

**Results:**

At 6 weeks, 203/257 (79%) HIV-infected infants were CMV-positive; 27 (11%) had congenital CMV, 108 (42%) postnatal CMV and 68 (26%) indeterminate timing of infection. By 6 months, 37/108 (34%) infants with postnatal CMV versus 16/54 (30%) CMV-negative infants died (adjusted hazard ratio (aHR) 1.1 [95%CI 0.6, 2.2]). At 6 weeks, 33/257 (13%) HIV-infected infants had EBV co-infection; 6 (2%) had congenital EBV, 18 (7%) postnatal EBV and 9 (4%) indeterminate timing of infection. By 6 months, 5/18 (28%) infants with postnatal EBV versus 72/224 (32%) EBV-negative infants died (aHR 0.8 [95%CI 0.3, 2.3]).

**Conclusions:**

The vast majority of HIV-infants had acquired CMV by 6 weeks, and EBV co-infection occurred earlier than expected, with one in eight HIV-infected infants positive for EBV by 6 weeks. There was a high prevalence of congenital CMV infection and we identified 6 infants with congenital EBV infection, which has not previously been reported in Africa or in the context of HIV infection. Neither CMV nor EBV co-infection was associated with increased mortality.

## Introduction

Despite scale-up of prevention of mother-to-child transmission (PMTCT) interventions, approximately 700 HIV-infected infants are born daily, predominantly in sub-Saharan Africa [1]. Without antiretroviral therapy (ART), mortality reaches 35% to over 50% during infancy and 67% by 2 years of age [2], [3]. In Zimbabwe, mortality between 2–6 months of age was 5-fold higher among HIV-infected compared to HIV-uninfected infants prior to ART [2].

The reasons for rapid disease progression remain incompletely understood. Advanced maternal disease [4], intrauterine infection [5] and high infant viral loads [6] have all been implicated. Co-infection with other viruses, such as cytomegalovirus (CMV) and Epstein Barr virus (EBV), has also been hypothesized to influence HIV disease progression [7], [8], [9]. CMV can be acquired *in utero*, or postnatally following exposure to breast milk or saliva [10]. In a US study of 52 HIV-infected infants, disease progression was more rapid in infants co-infected with CMV compared to those infected with HIV alone [8]. Postnatal CMV infection typically occurs earlier in sub-Saharan Africa than in Europe/USA [11], [12], [13]. However, no studies to date have investigated whether CMV influences HIV disease progression in sub-Saharan Africa, where CMV is acquired earlier and HIV disease course is more rapid than in USA/Europe.

HIV-infected children acquire EBV earlier in life than HIV-uninfected children, but in one prospective Canadian study there was no impact of EBV on HIV viral load or disease progression [14]. In Africa, primary EBV infection also occurs earlier and with higher peak viremia in HIV-infected compared to HIV-uninfected infants [15]. Furthermore, HIV-infected children are more likely to be co-infected with multiple EBV types, particularly prior to ART initiation [16]. However no studies to date have explored the impact of EBV on HIV disease progression in sub-Saharan Africa.

We investigated the frequency of co-infection with CMV and EBV in a cohort of HIV-infected infants born in Zimbabwe between 1997–2001, at the peak of the HIV epidemic, to test the hypothesis that early co-infection with either CMV or EBV was associated with increased mortality.

## Methods

### ZVITAMBO trial

ZVITAMBO was a randomized trial of vitamin A supplementation conducted between 1997–2001, as previously described [17]. 14110 mother-infant pairs were recruited within 96h of delivery, provided the infant was a singleton with no acute life-threatening condition and birth weight ≥1500 g, and the mother planned to stay in Harare after delivery. Follow-up was at 6 weeks and 3 months, then 3-monthly through 12–24 months. Plasma collected at recruitment and follow-up time-points was stored at −80C. Weight, height and head circumference were measured using standardized techniques [18]. Medical treatment and counseling regarding infant feeding were offered throughout the trial, which preceded availability of cotrimoxazole prophylaxis and ART.

### Maternal and infant HIV testing

At baseline, mothers were tested for HIV using two parallel ELISA assays, as previously described [17]. Absolute CD4 counts (FACScount; Beckton Dickinson) and viral loads (Roche Amplicor HIV-1 Monitor test v1.5) were measured in a subgroup of mothers at baseline (within 96 hours of parturition). Infants born to HIV-positive mothers were tested for HIV by DNA PCR within 96 h of birth and at 6 weeks to identify intrauterine and intrapartum infection, respectively. HIV viral load was measured in all HIV-infected infants with available samples at 6 weeks of age, as previously described [19].

### Study subjects

We designed a cross-sectional study of HIV-infected infants at 6 weeks of age, to test the hypothesis that early co-infection with either CMV or EBV was associated with increased mortality. We chose this time-point because the peak in early infant mortality in this cohort occurred between 2-6 months age [2]; we therefore reasoned that co-infection would occur prior to this mortality peak. The primary outcome of the study was mortality by 6 months of age.

With known 6-month mortality of 37% and estimating risk of death among infants with HIV/CMV co-infection to be 2.4-fold higher than among infants with HIV infection alone [8], and assuming CMV prevalence of 50% by 6 weeks, 330 infants would provide 80% power at 5% significance to reject the null hypothesis. We used simple random selection to identify HIV-infected infants with available plasma (50–100 µL) at 6 weeks of age.

### Detection of CMV and EBV by real-time PCR

Viral nucleic acid was isolated from 50–100 µL cryopreserved plasma using the QIAamp DSP Virus Spin Kit (Qiagen, Hilden). CMV and EBV were detected by real-time PCR on a LightCycler 2.0 instrument (Roche), using the Artus CMV LC and Artus EBV LC kits, respectively (Qiagen), with the manufacturer's recommended thermocycling conditions. Each run included an extraction control, amplification control and positive control. Nucleic acid extraction and PCR steps were done at separate laboratories to avoid contamination.

The manufacturer-specified limit of detection (LOD) for CMV and EBV was 0.65 and 5.78 copies/µL, respectively. Samples with values below these thresholds were considered negative; samples with values above the LOD were considered positive. The limit of quantification (LOQ) corresponded to the lowest standard supplied with the kit (10 copies/µL); this allowed us to quantify CMV and EBV viral loads>5000 copies/mL, based on our infant plasma volumes and assay elution volumes. Samples that were above the LOD but below the LOQ were considered ‘low positives’ [20].

For infants with positive CMV or EBV results at 6 weeks, a baseline sample (within 96h of birth) was retrieved, if available, to determine timing of infection. Infants were categorized as having congenital CMV or EBV infection if the birth sample was positive, or postnatal infection if the birth sample was negative but 6-week sample positive. Infants with positive results at 6 weeks but no available birth sample were categorized as having indeterminate timing of infection and excluded from analyses.

### Statistical methods

Characteristics were compared between groups using ANOVA or Kruskal-Wallis tests for continuous variables and Chi-squared test for categorical variables. Anthropometry measurements were converted to Z-scores using WHO Anthro version 3.0.1 (http://www.who.int/childgrowth/en). The effect of postnatal CMV or EBV acquisition on mortality from time of CMV/EBV detection (6 weeks of age) to death or 6 months was evaluated using Kaplan-Meier estimates. Because infants were selected at 6 weeks of age for inclusion in this study, we were not able to investigate the impact of congenital CMV or EBV infection on survival. Cox proportional hazard models were used to estimate hazard ratios for mortality, adjusting for maternal education, CD4 count and death, and infant gender, low birth weight and 6-week HIV viral load, which were risk factors for mortality in this cohort [2], [17], [19]. Statistical analyses were undertaken using STATA version 10 (Stata-Corp, College Station, TX, USA).

### Research Approvals

Mothers gave written informed consent for their own participation in the study, and written informed consent for participation of their infant. The original ZVITAMBO trial and this sub-study were approved by the Medical Research Council of Zimbabwe, Johns Hopkins Bloomberg School of Public Health, and Montreal General Hospital Ethics Committee.

## Results

### CMV co-infection

A total of 257 HIV-infected infants had sufficient archived plasma available at 6 weeks to be included in this study. At 6 weeks of age, 203/257 (79%) HIV-infected infants were CMV-positive. Baseline plasma (collected within 96h of birth) was available for 135/203 (67%) CMV-positive infants to ascertain timing of infection; 68 CMV-positive infants were therefore excluded from further analyses. Of the 135 infants tested at birth, 27 (20%) were CMV-positive and were classified as having congenital CMV (cCMV); the remaining 108 (80%) infants were classified as having postnatal CMV infection. Overall, therefore, at 6 weeks of age, 27 (11%) infants had cCMV, 108 (42%) postnatal CMV, 68 (26%) indeterminate timing of CMV infection and 54 (21%) were CMV-negative.

Characteristics of CMV-negative, cCMV and postnatal CMV groups are shown in Table 1. There were no significant differences in any maternal variable between groups. Infants with cCMV infection were significantly more likely to have intrauterine HIV infection (20/27; 74%), compared to CMV-negative (20/54; 37%) or postnatal CMV (39/108; 36%) groups (P = 0.001). There were no other significant differences in birth characteristics between groups. By 6 months of age, infants with cCMV had similar weight and length, but a trend towards lower head circumference Z-scores, compared to CMV-negative infants (mean(SD) −0.97(0.89) vs −0.33(1.20), respectively; P = 0.104; Table 1).

**Table 1:** Characteristics of mothers and infants by CMV status.

	Congenital CMV (n = 27)	Postnatal CMV (n = 108)	CMV Negative (n = 54)	P value
**Maternal factors** *				
Maternal age, years	25.7 (4.9)	25.5 (5.2)	25.6 (5.7)	0.99
Parity; median (IQR)	2 (1–3)	2 (2–3)	2 (1–3)	0.78
Married or stable union, n (%)	22 (82)	97 (91)	47 (87)	0.39
Education, years	10.3 (2.0)	9.6 (2.3)	9.5 (2.0)	0.27
Employed; n (%)	6 (22)	18 (17)	7 (13)	0.57
Family income, US dollars; median (IQR)	780 (548–2742)	1258 (684–2028)	1067 (493–1317)	0.16
MUAC, cm	25.0 (2.6)	26.1 (2.7)	25.5 (2.9)	0.13
Hemoglobin, g/L	101 (23) [21]	104 (20) [80]	109 (22) [31]	0.44
CD4 count, cells/mm^3^; median (IQR)	387 (189–480) [25]	313 (208–460) [89]	392 (223–575) [45]	0.33
**Infant Factors** *				
Male sex, n (%)	14 (52)	52 (48)	30 (56)	0.67
Vaginal delivery, n (%)	23 (85)	99 (92)	45 (83)	0.25
Gestational age, weeks	39.5 (0.9)	39.2 (1.5)	38.9 (1.6)	0.28
Intrauterine HIV infection; n (%)	20 (74)	39 (36)	20 (37)	
Intrapartum HIV infection; n (%)	7 (26)	69 (64)	34 (63)	0.001
Birth weight, grams	2931 (522)	2921 (440)	2827 (530)	0.46
Birth WAZ	−0.86 (1.15) [27]	−0.85 (1.02) [108]	−1.11 (1.24) [54]	0.36
6 week WAZ	−1.18 (1.26) [27]	−0.97 (1.47) [107]	−1.09 (1.75) [54]	0.78
3 Month WAZ	−1.56 (1.60) [19]	−1.39 (1.28) [74]	−1.69 (1.48) [38]	0.56
6 Month WAZ	−1.66 (1.84) [12]	−1.31 (1.46) [61]	−1.45 (1.56) [32]	0.76
Birth HAZ	−0.66 (1.29) [27]	−0.51 (1.28) [107]	−0.94 (1.47) [54]	0.17
6 week HAZ	−1.62 (1.42) [27]	−1.32 (1.35) [107]	−1.45 (1.77) [54]	0.60
3 Month HAZ	−1.23 (1.49) [19]	−1.57 (1.50) [74]	−1.65 (1.60) [39]	0.61
6 Month HAZ	−1.77 (1.58) [12]	−1.64 (1.38) [61]	−1.58 (1.52) [32]	0.93
Birth HCZ	−0.37 (1.19) [27]	−0.14 (1.11) [108]	−0.29 (1.48) [54]	0.62
6 week HCZ	−0.75 (1.10) [27]	−0.25 (1.24) [107]	−0.34 (1.42) [54]	0.19
3 Month HCZ	−0.81 (1.10) [20]	−0.24 (1.25) [74]	−0.62 (1.32) [40]	0.11
6 Month HCZ	−0.97 (0.89) [12]	−0.21 (1.38) [61]	−0.33 (1.20) [32]	0.19
Birth hemoglobin, g/L	175 (17) [21]	176 (21) [79]	180 (22) [31]	0.57
6 week hemoglobin, g/L	103 (17) [17]	105 (25) [60]	99 (21) [22]	0.65
6 week HIV viral load, log copies/mL	10.6 (1.8) [25]	10.5 (1.6) [104]	10.5 (1.6) [50]	0.94

*All values are mean (standard deviation), unless shown otherwise.

For variables with missing data, the number of participants with available data is shown in square brackets.

CMV: Cytomegalovirus; SD: standard deviation; MUAC: mid-upper arm circumference; WAZ: weight-for-age Z score; HAZ: height-for-age Z-score; HCZ: head circumference-for-age Z-score. P value is for comparison between three groups.

**Figure 1:**
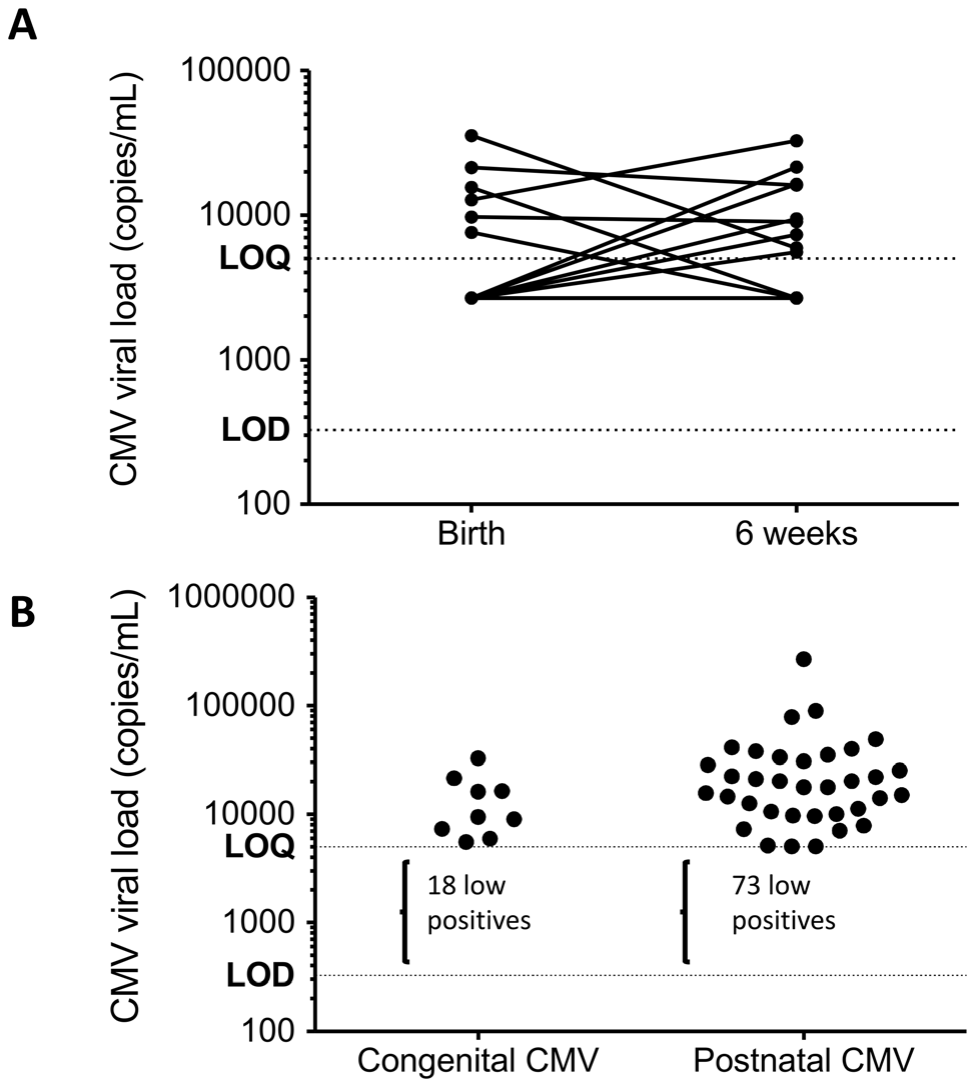
CMV viral loads in HIV-infected infants. A: CMV viral loads at birth (within 96 hours of delivery) and at 6 weeks of age in 27 infants with congenital CMV. Viral loads were measured following viral nucleic acid extraction from 100 uL plasma by real-time PCR, using the CMV Artus LC kit (Qiagen) on a LightCycler 2.0 (Roche) machine, with limit of detection of 0.65 copies/uL (equivalent to 325 copies/mL; shown as dotted line labeled LOD) and limit of quantification of 10 copies/uL (equivalent to 5000 copies/mL; shown as dotted line labeled LOQ). Infants with low positive results (above LOD but below LOQ) are shown arbitrarily as having a result halfway between the LOD and LOQ. B: Comparison of CMV viral loads at 6 weeks of age in 27 infants with congenital CMV and 108 infants with postnatal CMV. Infants with viral loads between LOD and LOQ are excluded because viral load could not be quantified, but numbers in each group with low-positive viral loads are shown.

CMV viral loads at birth were below the limit of quantification (5000 copies/mL) in 21/27 (78%) infants with cCMV; in 16 infants, viral loads remained below the limit of quantification (i.e. low positive) at 6 weeks, whilst in 5, the viral load increased above the limit of quantification by 6 weeks (Fig 1A). Among the 6 cCMV infants with quantifiable levels of CMV at birth, median (IQR) viral load was 14200 (9175, 24988) copies/mL, compared to 9450 (6640, 18975) copies/mL among cCMV infants with quantifiable viral load at 6 weeks of age (Fig 1A). Among infants with postnatal CMV, the majority (72/108; 67%) had viral load below the limit of quantification at 6 weeks (i.e. low positives); among those with quantifiable CMV, viral loads were median (IQR) 17800 (10050, 33600) copies/mL, which was not significantly different to viral loads in the cCMV group (P = 0.11; Fig 1B).

### Impact of CMV co-infection on mortality by 6 months

**Figure 2:**
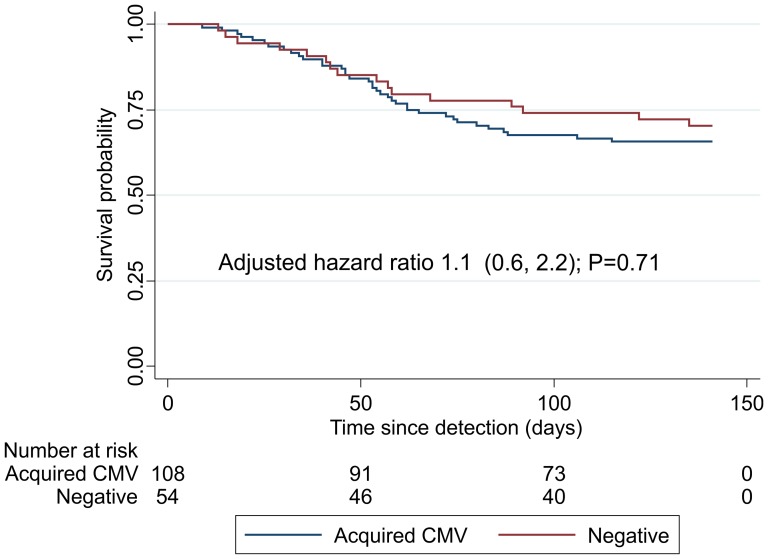
Survival in HIV-infected infants by postnatal CMV status. Survival among infants with postnatal CMV infection (negative CMV PCR at birth, positive CMV PCR at 6 weeks of age) or without CMV co-infection (negative CMV PCR at 6 weeks of age), from time of viral testing (6 weeks of age) through 6 months of age. Adjusted hazard ratio from Cox proportional hazards model is shown, adjusting for maternal education, CD4 count and death, and infant gender, low birth weight and 6-week HIV viral load.

37/108 (34%) infants with postnatal CMV died by 6 months of age, compared to 16/54 (30%) CMV-negative infants. The unadjusted hazard ratio for mortality was 1.2 (95%CI 0.7, 2.2) and, after adjusting for maternal education, CD4 count and death, and infant gender, low birth weight and 6-week HIV viral load, was 1.1 (95%CI 0.6, 2.2); Fig. 2. We repeated the analysis including all CMV-positive infants (n = 203) regardless of timing of CMV acquisition (congenital, indeterminate and postnatal infection) and found similar results (data not shown).

Taken together, therefore, the majority (79%) of HIV-infected infants were co-infected with CMV by 6 weeks of age; of those for whom precise timing of infection could be ascertained, one-fifth (20%) had congenital CMV. Postnatal CMV co-infection was not associated with increased mortality in HIV-infected infants.

### EBV co-infection

Of the 257 HIV-infected infants with archived plasma available, 33 (13%) had EBV co-infection at 6 weeks of age. Of these, 31/33 (94%) also had CMV co-infection. Birth samples were retrieved for 24/33 (73%) EBV-positive infants to ascertain timing of infection; 9 EBV-positive infants were therefore excluded from further analyses. Of the 24 infants tested at birth, 6 (25%) were EBV-positive and classified as having congenital EBV (cEBV); the remaining 18 (75%) infants were classified as having postnatal EBV infection. Of the 6 infants with cEBV infection, 2 (33%) also had congenital CMV infection. Overall, at 6 weeks of age, therefore, 6 (2%) infants had cEBV, 18 (7%) postnatal EBV, 9 (4%) indeterminate timing, and 224 (87%) were EBV-negative.

Characteristics of EBV-negative, cEBV and postnatal EBV groups are shown in Table 2. Mothers of infants with cEBV were younger than mothers of EBV-negative infants and had lower parity and CD4 count. There were no significant differences in infant characteristics at birth. By 6 weeks of age, infants with postnatal EBV had lower hemoglobin than EBV-negative infants (mean(SD) 84(30) vs 107(21) g/L, respectively; P = 0.001). There was a trend towards lower weight and length at 6 weeks in infants with congenital and postnatal EBV, compared to EBV-negative infants; by 6 months, head circumference tended to be lower in EBV-positive infants, although few infants had anthropometry data available.

**Table 2:** Characteristics of mothers and infants by EBV status.

	Congenital EBV (n = 6)	Postnatal EBV (n = 18)	EBV Negative (n = 224)	P value
**Maternal factors** *				
Maternal age, years	21.3 (3.1)	25.5 (3.7)	25.6 (5.2)	0.13
Parity; median (IQR)	1 (1–1)	2 (2–2)	2 (1–3)	0.03
Married or stable union, n (%)	5 (83)	15 (83)	202 (91)	0.49
Education, years	10.7 (1.0)	9.9 (1.5)	9.8 (2.2)	0.62
Employed; n (%)	3 (50)	1 (6)	41 (18)	0.05
Family income, US dollars; median (IQR)	1679 (661–2565)	733 (340–1354)	1186 (671–1902)	0.13
MUAC, cm	26.0 (1.1)	25.8 (1.9)	25.6 (3.0)	0.91
Hemoglobin, g/L	101 (27) [5]	105 (22) [14]	105 (20) [138]	0.90
CD4 count, cells/mm^3^; median (IQR)	239 (31–326) [6]	208 (129–419) [14]	341 (210–497) [191]	0.04
**Infant Factors** *				
Male sex; n (%)	4 (67)	10 (56)	113 (50)	0.68
Vaginal delivery; n (%)	4 (67)	16 (89)	199 (89)	0.25
Gestational age, weeks	39.2 (1.3)	38.8 (1.2)	39.2 (1.4)	0.46
Intrauterine HIV infection; n (%)	3 (50)	8 (44)	94 (42)	
Intrapartum HIV infection; n (%)	3 (50)	10 (56)	130 (58)	0.91
Birth weight, grams	3089 (594)	2931 (574)	2898 (456)	0.60
Birth WAZ	−0.55 (1.18) [6]	−0.87 (1.31) [18]	−0.91 (1.06) [222]	0.72
6 week WAZ	−1.60 (1.61) [6]	−1.56 (1.79) [17]	−0.96 (1.41) [224]	0.16
3 Month WAZ	−1.66(0.54) [3]	−1.82 (1.16) [12]	−1.45 (1.36) [156]	0.64
6 Month WAZ	−3.00 (0.00) [1]	−1.85 (1.56) [9]	−1.39 (1.48) [132]	0.39
Birth HAZ	−0.52 (1.16) [6]	−1.08 (1.01) [18]	−0.58 (1.36) [222]	0.31
6 week HAZ	−2.11 (0.62) [6]	−1.87 (1.45) [17]	−1.31 (1.48) [223]	0.15
3 Month HAZ	−1.45 (0.55) [3]	−2.36 (1.35) [12]	−1.36 (1.80) [158]	0.17
6 Month HAZ	−2.98 (0.00) [1]	−2.16 (1.39) [9]	−1.62 (1.38) [131]	0.34
Birth HCZ	0.02 (0.97) [6]	−0.16 (0.99) [18]	−0.18 (1.27) [224]	0.93
6 week HCZ	−0.31 (0.76) [6]	−0.22 (1.19) [17]	−0.28 (1.26) [223]	0.98
3 Month HCZ	−1.27 (0.04) [3]	−0.64 (1.27) [12]	−0.33 (1.23) [159]	0.31
6 Month HCZ	−0.77 (0.00) [1]	−0.76 (1.40) [9]	−0.23 (1.27) [132]	0.46
Birth hemoglobin, g/L	179 (14) [4]	177 (14) [14]	180 (24) [138]	0.89
6 week hemoglobin, g/L	101 (19) [3]	84 (30) [10]	107 (21) [104]	0.005
6 week HIV viral load, log copies/mL	10.4(1.0) [5]	10.8 (1.3) [18]	10.4 (1.8) [211]	0.75

*All values are mean (standard deviation), unless shown otherwise.

For variables with missing data, the number of participants with available data is shown in square brackets.

EBV: Epstein Barr virus; SD: standard deviation; MUAC: mid-upper arm circumference; WAZ: weight-for-age Z score; HAZ: height-for-age Z-score; HCZ: head circumference-for-age Z-score. P value is for comparison between three groups.

**Figure 3:**
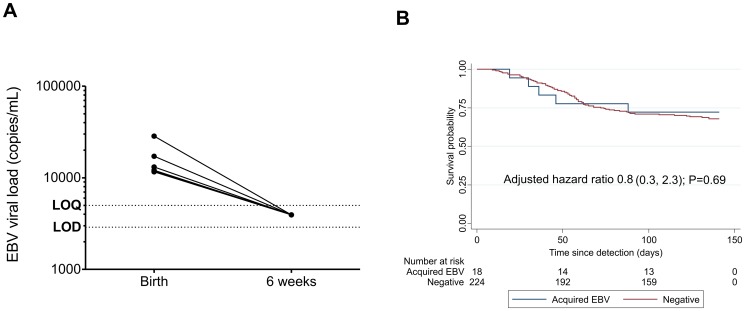
EBV viral loads and impact of EBV co-infection on survival in HIV-infected infants. A: Infants with congenital EBV had viral loads measured at birth (within 96 hours of delivery) and at 6 weeks of age. Viral loads were measured following viral nucleic acid extraction from 100 uL plasma by real-time PCR, using the EBV Artus LC kit (Qiagen) on a LightCycler 2.0 (Roche) machine, with limit of detection of 5.78 copies/uL (equivalent to 2890 copies/mL; shown as dotted line labeled LOD) and limit of quantification of 10 copies/uL (equivalent to 5000 copies/mL; shown as dotted line labeled LOQ). Infants with low positive results (above LOD but below LOQ) are shown arbitrarily as having a result halfway between the LOD and LOQ. B: Survival among infants with postnatal EBV infection (negative EBV PCR at birth, positive EBV PCR at 6 weeks of age) or without EBV co-infection (negative EBV PCR at 6 weeks of age), from time of viral testing (6 weeks of age) through 6 months of age. Adjusted hazard ratio from Cox proportional hazards model is shown, adjusting for maternal education, CD4 count and death, and infant gender, low birth weight and 6-week HIV viral load.

Among infants with cEBV infection, EBV viral load at birth was median (IQR) 12625 (11838, 20038) copies/mL; by 6 weeks of age, all these infants had EBV viral loads below the limit of quantification (Fig 3A). All infants with postnatal EBV at 6 weeks of age had low-positive viral loads, below the limit of quantification (<5000 copies/mL).

### Impact of EBV co-infection on mortality by 6 months

Although the study was not explicitly designed to evaluate the impact of EBV co-infection on mortality, we undertook exploratory analyses since we found a higher prevalence of EBV than expected. 5/18 (28%) infants with postnatal EBV died by 6 months of age, compared to 72/224 (32%) EBV-negative infants. The unadjusted hazard ratio for mortality was 0.9 (95%CI 0.4, 2.2) and, after adjusting for maternal education, CD4 count and death, and infant gender, low birth weight and 6-week HIV viral load, was 0.8 (95%CI 0.3, 2.3); Fig. 3B. We repeated the analysis including all EBV-positive infants (n = 33) regardless of timing of EBV acquisition (congenital, indeterminate and postnatal infection) and found similar results (data not shown).

Taken together, therefore, prevalence of EBV was higher than expected, with a substantial proportion (13%) HIV-infected infants already co-infected with EBV by 6 weeks of age; almost all these infants also had CMV co-infection. Among infants for whom precise timing of infection could be ascertained, one-quarter (25%) had congenital EBV infection. Infants with EBV co-infection at 6 weeks had lower hemoglobin and a trend towards poorer growth in early life. Postnatal EBV co-infection was not associated with increased mortality in HIV-infected infants, although the number of EBV-infected infants was small.

## Discussion

We show here that HIV-infected African infants become co-infected with herpesviruses early in life. In this cohort, the majority of infants had acquired CMV by 6 weeks of age and one-fifth of those for whom timing of infection could be ascertained had congenital CMV. In addition, EBV co-infection occurred much earlier than expected, with one-in-eight HIV-infected infants positive for EBV by 6 weeks; one-quarter of those for whom timing of infection could be ascertained had detectable EBV at birth, which has not commonly been reported, and never previously in sub-Saharan Africa. Neither CMV nor EBV co-infection were associated with increased mortality.

Overall, 79% infants were co-infected with CMV by 6 weeks of age. A similarly high CMV prevalence has been reported in previous, smaller cohorts of HIV-infected and uninfected African infants [21], [22], [23]. CMV acquisition is therefore almost ubiquitous during infancy in sub-Saharan Africa, compared with 10-40% prevalence in developed countries [8], [24]. Among HIV-exposed infants, transmission is related to breast milk CMV levels and maternal CD4 count [25]. The overall minimum estimate of congenital CMV prevalence in this cohort is 11%, but since timing of infection could not be ascertained for 68 infants, it is likely to be higher. Assuming similar prevalence among these 68 infants, the overall cCMV prevalence would be around 16%, which is plausible, given other reports from sub-Saharan Africa [23], [26]. Infants with birth weight <1500 g were excluded from the original ZVITAMBO trial, which may have led to a further underestimate of prevalence, because congenital CMV is associated with intrauterine growth restriction. cCMV prevalence is known to be higher in developing compared to developed countries [27], and among infants born to HIV-positive compared to HIV-negative mothers [7], [23], [24], [26]. Three-quarters of infants with cCMV infection also acquired HIV *in utero*, suggesting shared risk factors for intrauterine transmission. *In vitro*, HIV replication is up-regulated in syncytiotrophoblast cells of the placenta by simultaneous co-infection with CMV [28]. HIV infection may impact the clinical course of cCMV. HIV-infected newborns had a threefold higher risk of developing symptomatic cCMV compared to HIV-uninfected newborns with cCMV in the French Perinatal Cohort [24]. Infants in our study were not systematically examined at birth so information on symptomatic cCMV was not available. However, infants with cCMV tended to have reduced head circumference by 6 months. Among infants born to HIV-infected mothers in the Zambian CIGNIS cohort, those who were CMV-positive similarly had reduced head circumferences and lower psychomotor development compared to CMV-negative infants [29]. Thus the dual burden of HIV and CMV makes these infants highly vulnerable to poor neurodevelopmental outcomes.

Our study showed no impact of either postnatal CMV or EBV on infant mortality. *In vitro* studies provide conflicting data on whether CMV stimulates or inhibits HIV replication [30], [31]. One prior US study showed higher CMV antibody titers in children with symptomatic compared to asymptomatic HIV infection, suggesting a role for CMV as a co-factor in disease progression [32]. Similarly, among HIV-infected infants born in the US, those co-infected with CMV had lower CD4 counts [7] and increased mortality compared to those infected with HIV alone [8]. Several studies have reported CMV as a risk factor for disease progression in adults both before and after ART [33], [34]. By contrast, in a cohort of 81 Spanish HIV-infected infants, there was no difference in mortality or immunosuppression between those with and without positive CMV urine cultures [35]. We found almost universal CMV acquisition in early infancy, irrespective of outcome, although we were unable to ascertain the contribution of symptomatic CMV disease to mortality.

The impact of EBV co-infection has received little attention in HIV-infected infants. Children in sub-Saharan Africa are known to acquire EBV earlier than in Europe/USA [15], [36], [37], [38], [39]. Studies of HIV-uninfected and HIV-infected infants in Kenya have shown substantial acquisition of EBV before 6 months of age [15], [38], whilst another Kenyan study [39] of HIV-infected infants reported 14% EBV prevalence at 3-4 months of age. We found a similar proportion (13%) of HIV-infected infants had EBV co-infection at 6 weeks of age. These findings challenge the common assumption that EBV is only acquired later in infancy, once maternal anti-EBV antibodies have waned [40]. Kenyan HIV-infected infants acquired EBV earlier than HIV-uninfected infants, had higher peak EBV viremia and more severe symptoms, including hospitalization [15]. It was striking that 31 of 33 infants with detectable EBV at 6 weeks of age also had CMV co-infection, suggesting shared risk factors for transmission. CMV and EBV co-infection have been described in several settings among adults [41], [42] and children [43], [44], [45], and interactions between these viruses appear to affect immune function [46], [47], [48]. It was not therefore possible to distinguish the impact of EBV acquisition from CMV acquisition in this current study, since almost all infants were co-infected.

A particularly novel finding in our study was the detection of EBV infection at birth in several HIV-infected infants. At a minimum, the prevalence of EBV at birth was 6/257 (2%), although 9 infants who were EBV-positive at 6 weeks had no birth sample available to determine timing of infection. Furthermore, we only tested birth samples if we detected EBV at 6 weeks of age, which assumes that infants infected at birth remained viremic at 6 weeks. Since birth samples were not available for many infants it was not possible to more systematically test all infants at birth. For these reasons, it is possible that the prevalence of EBV at birth was actually higher than reported here. We think it is most likely that detection of EBV in plasma at birth indicates congenital infection, although further studies are needed to confirm transmission from mother to child; to explore the precise timing of vertical infection (intrauterine vs. intrapartum); to isolate virus by other methods, such as culture, from plasma and other compartments; and to identify infant immune responses to EBV soon after birth (e.g. IgM antibodies to the viral capsid antigen). Congenital EBV has not been well characterized to date. A small number of case reports and a case series from the 1970s found that some infants were asymptomatic whilst others had widespread hematologic and hepatic disturbances at birth [49], [50], [51], [52]. In some cases, co-infection with cCMV was identified [50]. In our cohort, 2/6 infants with cEBV also had cCMV infection. More recently, an Australian case-control study reported 0.9% cEBV prevalence in children with cerebral palsy (n = 339) compared to 0% in healthy controls (n = 594) [53]. However we could find no previous reports of cEBV in the context of HIV infection or from sub-Saharan Africa. In our study, infants with cEBV had similar birth characteristics to other infants; however, their mothers were younger and more immunosuppressed. EBV-infected children (irrespective of timing of infection) were more anemic and had mild growth impairment compared to EBV-uninfected children; however, there were limited data on clinical outcomes. Given the substantial 2% prevalence reported here, further studies are needed to ascertain whether cEBV occurs in other cohorts, particularly in the PMTCT era, and to determine the consequences of congenital infection; for example, whether these infants are at elevated risk of EBV-driven malignancy [38].

This study has several strengths and some weaknesses. We drew specimens and clinical data from a unique natural history cohort, in which well-characterized mother-infant pairs were recruited prior to scale-up of ART and followed closely to ascertain mortality. Although this is the largest study of infant CMV/HIV co-infection to date from sub-Saharan Africa, our final sample size was smaller and the CMV prevalence higher than we estimated, and we may therefore have been underpowered to determine a true mortality effect. The study was not designed or powered to evaluate the impact of EBV co-infection on mortality, but because we found higher than expected EBV prevalence at 6 weeks, we undertook exploratory analyses. The absence of any detectable effect on mortality may therefore be due to the small number of EBV cases. Infants had to survive to 6 weeks to be included in this study, which introduced selection bias by design. Competing risks prior to the 6-week evaluation may have limited our ability to detect an association between viral co-infection and mortality; however, there were very few deaths among HIV-infected infants before 6 weeks. We used molecular techniques to detect CMV and EBV viremia and did not have available archived samples to undertake serological testing or to evaluate other compartments; however, it is quite likely that some infants infected with CMV or EBV had absent DNA in plasma [15]. Furthermore, the limit of detection of the PCR assays used may have led to some infants who were viremic being wrongly classified as uninfected. Together, these factors would have led us to underestimate the prevalence of CMV and EBV at birth and 6 weeks of age, which may have contributed to the null association between co-infection and mortality.

In conclusion, HIV-infected infants in sub-Saharan Africa are highly susceptible to early co-infection with herpesviruses. The vast majority are co-infected with CMV by 6 weeks of age; at least 11% of HIV-infected infants had congenital CMV infection. EBV infection is acquired much earlier in life than previously assumed, with one-in-eight infants EBV-positive by 6 weeks of age; at least 2% of HIV-infected infants had congenital EBV infection. This study highlights the vulnerability of HIV-infected infants to persistent viral infections and calls for further research to evaluate the consequences of congenital and postnatal co-infections in this population.
